# Heme Competition Triggers an Increase in the Pathogenic Potential of *Porphyromonas gingivalis* in *Porphyromonas gingivalis-Candida albicans* Mixed Biofilm

**DOI:** 10.3389/fmicb.2020.596459

**Published:** 2020-12-02

**Authors:** Yanyang Guo, Yu Wang, Yijin Wang, Yabing Jin, Chen Wang

**Affiliations:** Jiangsu Key Laboratory of Oral Diseases, Department of Prosthodontics, Affiliated Hospital of Stomatology, Nanjing Medical University, Nanjing, China

**Keywords:** Porphyromonas gingivalis, periodontitis, Candida albicans, heme competition, gingipains

## Abstract

As one of the main pathogens of periodontitis, *Porphyromonas gingivalis* often forms mixed biofilms with other bacteria or fungi under the gingiva, such as *Candida albicans*. Heme is an important iron source for *P. gingivalis* and *C. albicans* that supports their growth in the host. From the perspective of heme competition, this study aims to clarify that the competition for heme enhances the pathogenic potential of *P. gingivalis* during the interaction between *P. gingivalis* and *C. albicans*. *Porphyromonas gingivalis* single-species biofilm and *P. gingivalis*-*C. albicans* dual-species biofilm were established in a low- and high-heme environment. The results showed that the vitality of *P. gingivalis* was increased in the dual-species biofilm under the condition of low heme, and the same trend was observed under a laser confocal microscope. Furthermore, the morphological changes in *P. gingivalis* were observed by electron microscope, and the resistance of *P. gingivalis* in dual-species biofilm was stronger against the killing effect of healthy human serum and antibiotics. The ability of *P. gingivalis* to agglutinate erythrocyte was also enhanced in dual-species biofilm. These changes disappeared when heme was sufficient, which confirmed that heme competition was the cause of thepathogenicy change in *P. gingivalis*. Gene level analysis showed that *P. gingivalis* was in a superior position in the competition relationship by increasing the expression of heme utilization-related genes, such as HmuY, HmuR, HusA, and Tlr. In addition, the expression of genes encoding gingipains (Kgp, RgpA/B) was also significantly increased. They not only participate in the process of utilizing heme, but also are important components of the virulence factors of *P. gingivalis*. In conclusion, our results indicated that the pathogenic potential of *P. gingivalis* was enhanced by *C. albicans* through heme competition, which ultimately promoted the occurrence and development of periodontitis and, therefore, *C. albicans* subgingival colonization should be considered as a factor in assessing the risk of periodontitis.

## Introduction

Periodontitis is one of the main causes of tooth loss (Hajishengallis et al., 2012). As the sixth largest infectious disease in the world, it is also closely related to systemic diseases such as rheumatoid arthritis, atherosclerosis, and Alzheimer’s disease (Kumar, 2013; Kassebaum et al., 2014; Hussain et al., 2015; Leech and Bartold, 2015; Singhrao et al., 2015). Periodontitis is caused by subgingival plaque biofilm, which is rich in abundant and diverse microorganisms (Flemming and Wingender, 2010; Valm, 2019). Research on the relationship between microorganisms in biofilm is of great significance for revealing the pathogenic principle of subgingival biofilm, and will also guide clinical practice to a certain extent.

The detection rate of *Porphyromonas gingivalis*, one of the major pathogens of periodontitis in subgingival biofilm, is as high as 60.9% (Nagano et al., 2018). In recent years, it has been found that *Candida albicans*, a pathogen of denture stomatitis, also has an increased frequency of subgingival separation in patients with periodontitis (McManus et al., 2012; Sardi et al., 2012; Marsh and Zaura, 2017). It can form a mixed biofilm with *P. gingivalis*. Previous studies using animal models of periodontal disease have shown that dual-species biofilm is more pathogenic than single-species biofilm (Daep et al., 2011). This increase in pathogenicity may be due to the formation of more complex and orderly microbial communities or the interaction between microorganisms to promote the secretion of virulent proteins, which will increase the difficulty of clinical treatment (Peleg et al., 2010; Harriott and Noverr, 2011; Xu and Dongari-Bagtzoglou, 2015; Hajishengallis and Lamont, 2016; Rajendran et al., 2016). However, at present, research on the dual-species biofilm formed by *P. gingivalis* and *C. albicans* is mostly confined to the influence of characterization changes, and we know little about their interplay mechanism. Therefore, further exploration of the molecular mechanisms of the interaction between *P. gingivalis* and *C. albicans*, especially the influence of *C. albicans* on periodontal pathogen *P. gingivalis*, will be conducive to a deepened understanding of the pathogenesis of periodontitis.

Heme, an iron porphyrin compound containing ferrous ion, is an important iron source for many microorganisms, such as *P. gingivalis* and *C. albicans* (Olczak et al., 2005; Roy and Kornitzer, 2019). *Porphyromonas gingivalis* has a mature system for the uptake and utilization of heme, including secreting protease to degrade hemoglobin to release heme and globin, and then transporting heme into bacteria for utilization through transmembrane protein receptors (Olczak et al., 2005; Slezak et al., 2020). Nutrients such as heme are not always abundant, and in the absence of nutrients, different microorganisms react differently. Some microorganisms choose to reduce their own nutrient requirements, some use other alternative pathways such as metabolic changes, and others take absolute ownership of nutrients by killing other competitors (Miramon and Lorenz, 2017; Ganas and Schwendicke, 2019). Trejo-Hernandez et al. (2014) showed that *C. albicans* and *Pseudomonas aeruginosa* compete for iron, which determines the process and severity of mixed infection. Because *P. gingivalis* and *C. albicans* can exist in the same niche and *P. gingivalis* has a more complete system of heme utilization, we suspect that heme competition occurs between *P. gingivalis* and *C. albicans*, and *P. gingivalis* is in a dominant position in this competition.

In this study, we explored for the first time the role of heme competition between *P. gingivalis* and *C. albicans*, and it provides a new method to study the crosstalk between bacteria and fungus. Our study reveals that the existence of multi-species biofilm is often more pathogenic than single-species biofilm.

## Materials and Methods

### Microbial Culture

*Porphyromonas gingivalis* ATCC33277 was grown on brain heart infusion (BHI; OXOID, Basingstoke, England) agar supplemented with 0.1 mg L^−1^ vitamin K (Bomei, Hefei, China) and 5 mg L^−1^ hemin (Sigma, St. Louis, MO, United States) or in BHI liquid medium under anaerobic condition at 37°C. *Candida albicans* SC5314 was aerobically cultured in Sabouraud dextrose agar (SDA; Hopebio, Qingdao, China) and then in SDA liquid medium agitated at 37°C. Microorganisms were collected *via* centrifugation of bacteria (5000 × *g*, 5 min) or fungi (3000 × *g*, 5 min), and washed three times with phosphate buffered saline (PBS; HyClone, Logan, UT, United States). These microorganisms were used for the next interaction experiments.

### Models of Single- and Dual-Species Biofilm Formation

Roswell Park Memorial Institute (RPMI) 1640 medium (Gibco, NY, United States) was used as the coculture medium, with the addition of 0.1 μg/ml heme to construct a limited heme environment, and 5 μg/ml heme for an excessive heme environment (Dashper et al., 2009; Veith et al., 2018). To build a dual-species biofilm, 1 ml *C. albicans* solution (2 × 10^6^/ml) and 1 ml *P. gingivalis* solution (2 × 10^8^/ml) were cocultured of 24 h in 12-well plates under a hypoxic environment that conforms to the subgingival microenvironment. For single-species biofilm, 2 ml 1 × 10^8^ *P. gingivalis* solution or 1 × 10^6^ *C. albicans* solution was cultured in the same environment as previously described. The 12-well plates were placed in the anaerobic station (BUGBOX-M, Bridgend, England) for 10 min for degassing, and then wrapped with Parafilm to create a hypoxic environment (Trejo-Hernandez et al., 2014). The cultures were incubated at 37°C without shaking.

### Population Dynamics Measurement

The dynamic changes in bacteria and fungus were determined by counting the number of colony-forming units (CFUs) ml^−1^. Specifically, the dual-species biofilm and two types of single-species biofilm were gently washed twice with PBS, then collected in Eppendorf tubes. The samples were diluted using the method of serial dilution. Finally, 100 μl diluent of each sample was transferred onto SDA and BHI plates and coated on the surface with a glass rod. *Candida albicans* and *P. gingivalis* were cultured as previously described in section “Microbial Culture.” The number of colonies was counted using a bacterial colony counter.

### Biofilm Staining and Visualization With a Laser Scanning Confocal Microscope

In order to observe the changes in microbial vitality more clearly, biofilms were dyed with live/dead stain (L7012, Invitrogen, United States). Single- or dual-species biofilms were observed with a Zeiss LSM 710 laser scanning confocal microscope (Carl Zeiss Meditec AG, Jena, Germany) with a 10× and 40× oil immersion objective. Biofilms were formed on circular slides at the bottom of 12-well plates, washed twice with sterile PBS to remove microorganisms that did not form the biofilm, and then the live/dead staining liquid was mixed and added into the well plates. The mixtures were incubated for 15 min in the dark, and excess dye solution was removed. Sterile filter paper strips were used to remove all the remaining moisture and the samples were viewed under the excitation wavelengths of 488 and 561 nm. Three-dimensional images of biofilm were analyzed by ZEN Lite Microscopy Software.

### Scanning Electron Microscopy Observation of Biofilms

The preparation methods of the biofilms were as described above, and then, the biofilms were dehydrated with ethanol solutions of different concentrations. After spraying the microorganisms on biofilm with gold, their microscopic morphology was observed under a scanning electron microscope (SEM; TESCAN MAIA3, Czech Republic).

### Escape From Serum Killing Ability and Drug Resistance

Percoll density gradient centrifugation and ultrasonic oscillation were used to separate *P. gingivalis* and *C. albicans* from dual-species biofilm (Trejo-Hernandez et al., 2014). Additional details are found in the Supplementary Material. The serum used in the experiment was from healthy volunteers from the School of Stomatology, Nanjing Medical University, and its use was approved by the ethics committee of Nanjing Medical University (PJ2020-024-001). *Porphyromonas gingivalis* solution in two biofilms with a concentration of 1 × 10^8^/ml was diluted with BHI medium to a ratio of 1: 10, and then, 75 μl of bacteria solution and 25 μl of serum were added to a 96-well plate, which was anaerobically incubated at 37°C for 1 h. Serum was incubated in a 56°C water bath for 30 min and then used as the negative control. The bacterial liquid was continuously diluted, the same gradient of diluted bacterial liquid was coated on a BHI plate, and the plates were then anaerobically incubaed at 37°C for 7 days. The CFU were recorded with a colony meter to calculate the survival rate of each strain after serum treatment.

*Porphyromonas gingivalis* separated from single- and dual-species biofilm was adjusted to 1 × 10^8^/ml, and then, 100 μl bacterial liquid was coated on a BHI plate and immediately spread. At the same time, 6-mm diameter cefazolin (30 μg/tablet) and sulfamethoxazole (300 μg/tablet) drug-sensitive paper sheets were pasted on the plate. After 7 days of anaerobic culture at 37°C, the inhibition zone was measured.

### Hemagglutination Activity Assay

*Porphyromonas gingivalis* in single- and dual-species biofilms was separated by Percoll density gradient centrifugation and adjusted to 1 × 10^8^/ml. Additional details are provided in the Supplementary Material. First, 100 μl of each bacterial suspension was added to the first well of a 96-well plate with a U-shaped bottom. After that, each suspension was serially diluted in half, by taking 50 μl bacterial suspension from the former well and mixing it with 50 μl PBS (1:2 dilution). This step was repeated until a 1:128 dilution was achieved. Next, 100 μl 1% sheep red blood cell suspension was added to each well, and the plate was incubated for 3 h at room temperature. The agglutination of erythrocyte was observed, and the reciprocal of the maximum dilution multiple of the bacterial liquid with positive agglutination of erythrocytes was recorded.

### Quantitative Real-Time PCR

After 24 h of biofilm formation, the total RNA of *P. gingivalis* in single- and dual-species biofilm was extracted by use of a kit (B518625, Shanghai Sangon Biotech, China) in according to the protocol provided by the manufacturer. For complementary DNA (cDNA) synthesis, 1.0 μg RNA of each sample was amplified using a T3 theemocycler (Mastercycler 5333, Eppendorf, Hamburg, Germany) and the PrimeScript 1st Strand cDNA Synthesis kit (TaKaRa, Tokyo, Japan). Quantitative PCR was performed using the FastStart universal SYBR Green Master (ROX) kit (Roche, Basel, Switzerland) and the quantitative real-time amplification system (7900HT Fast, Applied Biosystems, Foster City, CA, United States). PCR amplification was performed using the primers specified in Table 1. The expression levels of mRNA of the target genes were normalized to the 16sRNA gene and calculated according to the 2^-*Δ*ΔCt^ method.

**Table 1:** Primers used for real time PCR (RT-PCR).

Target gene	Forward primer sequence (5'-3')	Reverse primer sequence (5'-3')
HagA	TAAATAAGGGCGGAGCAAGA	GACGGAAAGCAACATACTTCG
HmuY	GGCTACTACCGTTCCGACAG	ATCCCTGTGCGTTCTTCTTG
HmuR	CTCCCATGCGGCCAACCCTCC	GCAGACGGGCTGTACGGCTACC
HusA	CACACTGGCTGCGGACTT	CCGCTTTGCTCAGCATGGAT
Tlr	CCTGCGGGAACGGACAATATC	GCTACCGCCGAAGAGAGAAAC
Kgp	AGGAACGACAAACGCCTCTA	GTCACCAACCAAAGCCAAGA
RgpA	CACCGAAGTTCAAACCCCTA	GAGGGTGCAATCAGGACATT
RgpB	GCTCGGTCAGGCTCTTTGTA	GGGTAAGCAGATTGGCGATT
16sRNA	AGGCAGCTTGCCATACTGCG	ACTGTTAGCAACTACCGATGT

### Statistical Analysis

All experiments were independently performed at least three times. The data were analyzed by SPSS 24.0 software (SPSS Inc., Chicago, IL, United States) to test whether there were differences between single- and dual-species biofilms. All data are expressed as the mean ± SD of three separate experiments. Statistically significant differences (*p* < 0.05) were determined *via* one-way ANOVA.

## Results

### Population Dynamics of *Candida albicans* and *Porphyromonas gingivalis*

**Figure 1:**
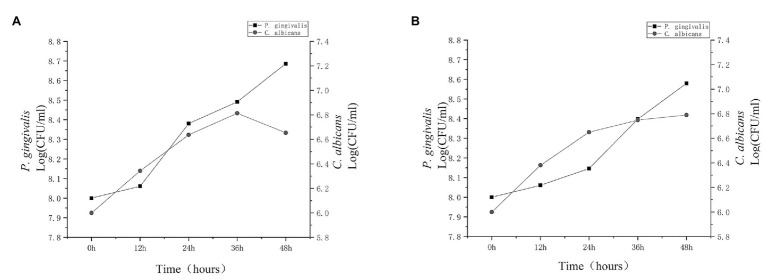
Population dynamics of *Candida albicans* and *Porphyromonas gingivalis*. *Porphyromonas gingivalis* and *C. albicans* single-species biofilms and *P. gingivalis*-*C. albicans* dual-species biofilm were cultured for 48 h. Growth dynamics of *C. albicans* (circles) and *P. gingivalis* (squares) were observed in **(A)** cocultures and **(B)** monocultures for 12, 24, 36, and 48 h. Growth was based on colony-forming units (CFU). After 36 h, the activity of *C. albicans* in the dual-species biofilm clearly decreased.

In order to understand the interaction between *C. albicans* and *P. gingivalis*, we first analyzed the viability of the two types of microorganisms at 12, 24, 36, and 48 h under hypoxia with 0.1 μg/ml heme. As shown in Figure 1, the presence of *P. gingivalis* did not affect *C. albicans* during the first 36 h, However, after 36 h, the number of *C. albicans* in the dual-species biofilm gradually decreased, unlike in the single-species biofilm. Additional details are provided in the Supplementary Material. The growth dynamics of *P. gingivalis* were similar between monocultures and cocultures, and to some extent, the presence of *C. albicans* increased the population of *P. gingivalis*.

**Figure 2:**
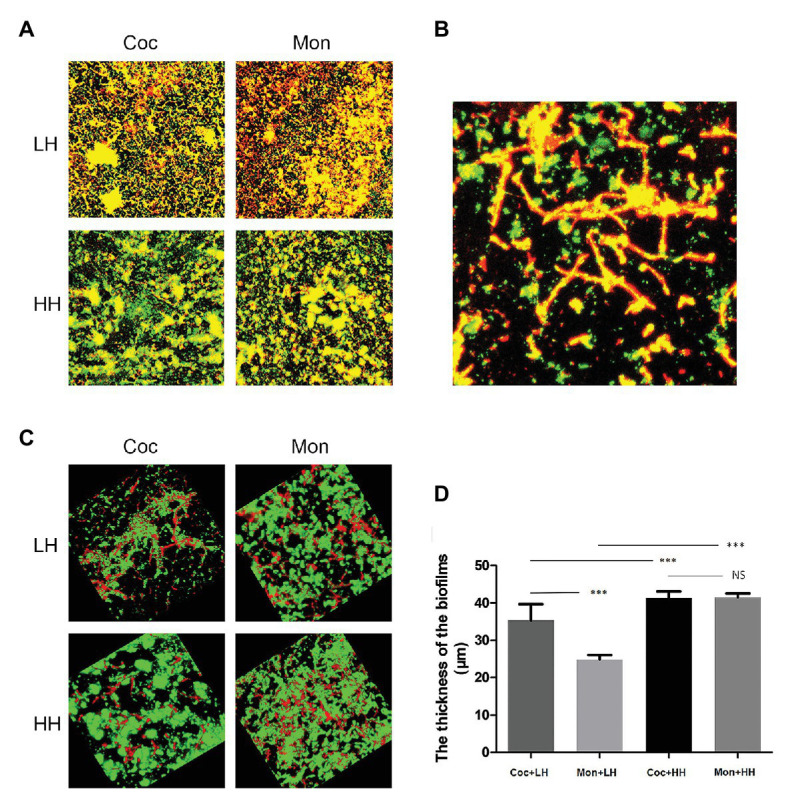
Epifluorescence images of biofilms in low-heme and high-heme environments. *Porphyromonas gingivalis* single-species biofilm and *P. gingivalis*-*C. albicans* dual-species biofilm were cultured for 24 h. The biofilms were stained with the LIVE/DEAD staining system. Red cells (stained with propidium iodide) are considered dead, while live cells remained green (stained with SYTO9). **(A)** 2D images of biofilms after 24 h. **(B)** Dead *C. albicans* in dual-species biofilm under a low-heme environment. **(C)** 3D images of biofilms after 24 h. **(D)** The thicknesses of biofilms were measured and analyzed by ZEN Lite Microscopy Software. ^***^p < 0.001; NS *p* > 0.05. LH, low heme; HH, high heme; Coc, coculture; and Mon, monoculture.

Combined with the growth trends of the two microorganisms, *P. gingivalis* seems to be able to kill *C. albicans* after 36 h of co-culture. We stained the biofilm of *P. gingivalis* and the dual-species biofilm. In view of the above hypothesis of heme competition, we stained biofilms in the high-heme environment for comparison. The number of living *P. gingivalis* in the dual-species biofilm was increased compared with that of the single-species biofilm, while it was observed that *C. albicans* was death. Under the condition of sufficient heme, the number of living *P. gingivalis* and *C. albicans* significantly increased (Figures 2A,B).

The three-dimensional images reveal that in dual-species biofilm, *P. gingivalis* was mainly green (living bacteria), while *C. albicans* was mainly red (dead fungus). In single-species biofilm, there was a clear increase in dead *P. gingivalis* compared with the dual-species biofilm. Under the condition of high heme, the green areas of single- and dual-species biofilm were obviously increased, suggesting that the number of living microorganisms was increased (Figure 2C).

The measurement of biofilm thickness showed that the biomass of dual-species biofilm was greater than that of single-species biofilm under the condition of low heme. Under the condition of high heme, there was no obvious difference in the thicknesses of the two types of biofilms, but all were greater than those under the condition of low heme (Figure 2D).

### SEM Examination of Biofilms

**Figure 3:**
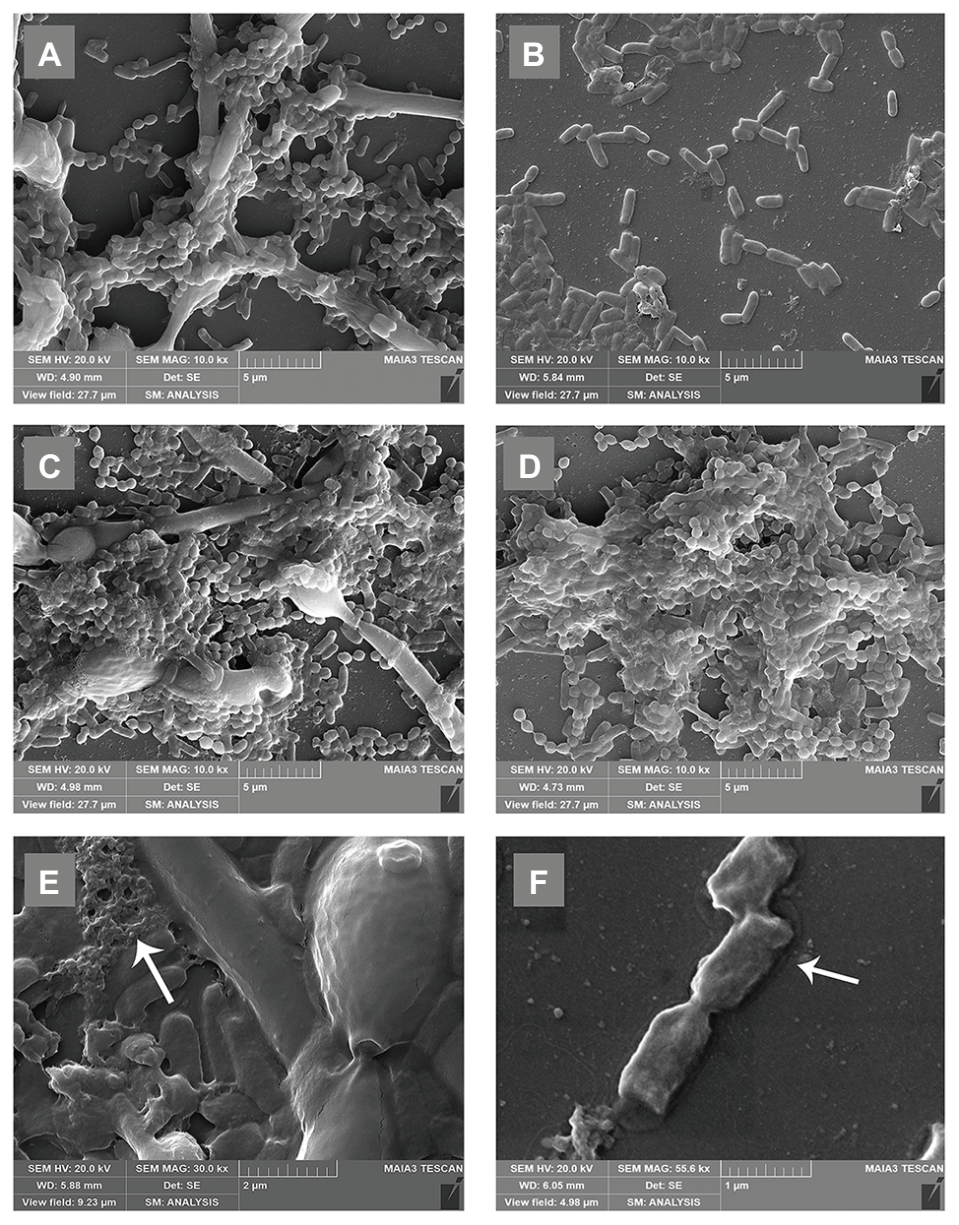
Scanning electron microscopy (SEM) microtopographies of biofilms in low-heme and high-heme environments. Biofilms in low-heme and high-heme environments were cultured for 24 h. **(A)** *Porphyromonas gingivalis*-*C. albicans* dual-species biofilm under low-heme condition. **(B)** *Porphyromonas gingivalis* single-species biofilm under low-heme condition. **(C)** *Porphyromonas gingivalis*-*C. albicans* dual-species biofilm under high-heme condition. **(D)** *Porphyromonas gingivalis* single-species biofilm under high-heme condition. **(E)** Under a low-heme environment, a large amount of exopolysaccharides (EPS; white arrow) was observed in the dual-species biofilm (×30.0 k). **(F)** Shrinking dead bacteria (white arrow) were discovered in *P. gingivalis* single-species biofilm under a low-heme environment (×55.6 k).

To further understand the effects of *C. albicans* on the morphology of *P. gingivalis* biofilms, we observed the biofilms with a scanning electron microscope. In the dual-species biofilm, *C. albicans* exhibited obvious hyphae, and the short rod shape of *P. gingivalis* was connected with *C. albicans* hyphae (Figure 3A). In addition, a large amount of exopolysaccharides (EPS) around *C. albicans* was observed in the dual-species biofilm, and it was wrapped around *C. albicans* and *P. gingivalis* to protect them against external stimulation (Figure 3E). Compared with single-species biofilm, the microorganisms in the dual-species biofilm overlapped each other and formed a multi-layer structure (Figure 3A). In the single-species biofilm, *P. gingivalis* changed to a rod shape, and the biofilm consisted of a single layer of bacteria and few EPS (Figure 3B). At the same time, we also noted some dead bacteria in the single-species biofilm that shrank and were prone to rupture (Figure 3F). Under the condition of sufficient heme, the morphological differences of *P. gingivalis* in single- and dual-species biofilms were reduced. Both were short rods, and the biomass was also significantly increased (Figures 3C,D).

### The Ability to Escape Host Immunity and Antibiotic Killing

**Figure 4:**
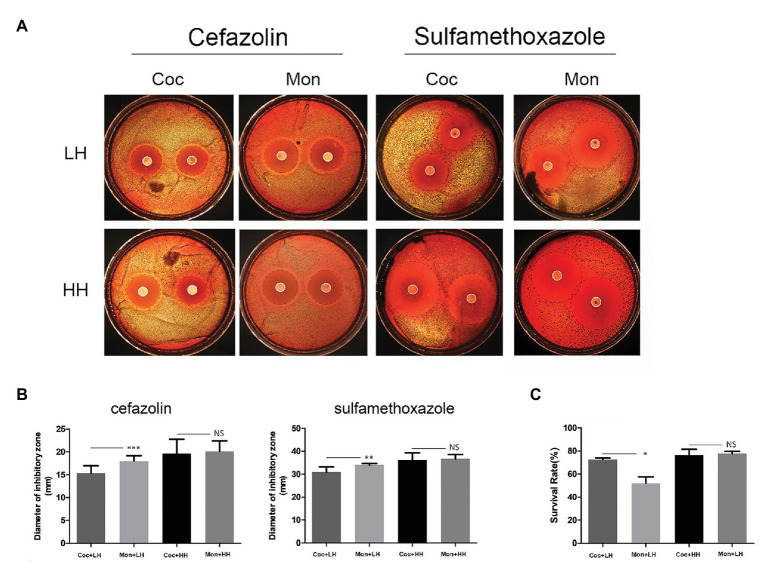
Ability of *P. gingivalis* to escape antibiotic and serum killing effects. **(A)** *Porphyromonas gingivalis* was separated and coated on brain heart infusion (BHI) plates. For the assay, 6-mm diameter cefazolin (30 μg/tablet) and sulfamethoxazole (300 μg/tablet) drug-sensitive paper was placed on BHI plates for 7 days. The black dotted line indicates the inhibition zone. **(B)** The diameters of the inhibition zone were measured and compared. **(C)** After co-culturing with serum for 1 h, the survival rates was compared. Viable counts were based on CFU. The calculation formula is as follows: survival rate (%) = (number of bacteria surviving after fresh serum co-incubation/number of bacteria surviving after inactivated serum co-incubation) × 100%. ^*^p < 0.05; ^**^p < 0.01; ^***^p < 0.001; NS *p* > 0.05. LH, low heme; HH, high heme; Coc, coculture; and Mon, monoculture.

To survive in the body, bacteria must resist the killing ability of serum and the pressure of antibiotics. We used the disk diffusion method to detect the sensitivity of *P. gingivalis* to cefazolin and sulfamethoxazole in the biofilms of single and double species. The diameters of the inhibition zones of cefazolin and sulfamethoxazole in the dual-species biofilm were 15.32 ± 1.67 mm and 30.93 ± 2.26 mm, respectively, which were smaller than those of single-species biofilm (Figures 4A,B).

Among the single-species biofilm, the survival rate of *P. gingivalis* was only 52.1% after treatment with healthy serum. After forming dual-species biofilm with *C. albicans*, the survival rate of *P. gingivalis* increased to 72.8% (*p* = 0.029), with statistically significant difference (Figure 4C). When heme was sufficient, the above differences of *P. gingivalis* in the two biofilms disappeared (Figures 4A–C). Additional details are provided in the Supplementary Material.

### The Capacity for Erythrocyte Agglutination of *Porphyromonas gingivalis*

**Figure 5:**
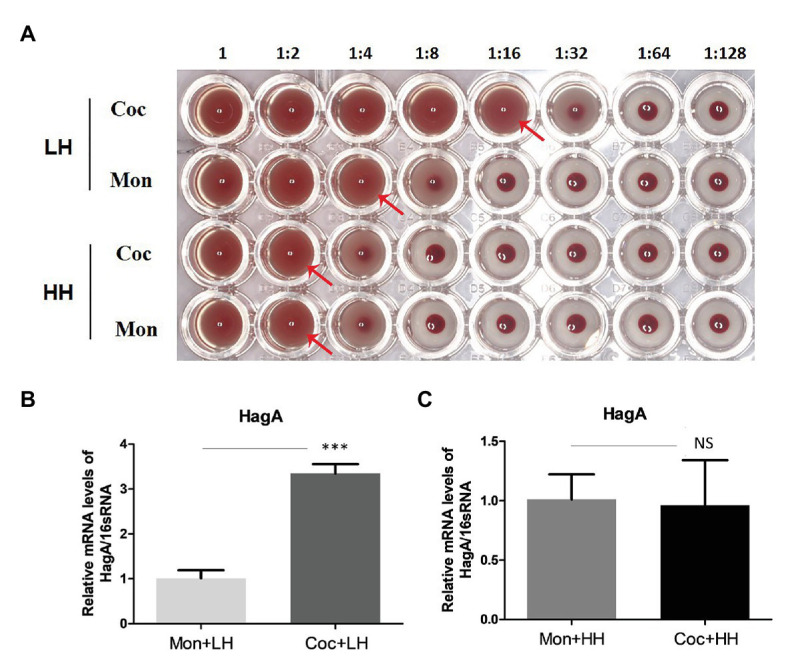
Hemagglutination activity of *P. gingivalis*. **(A)** Hemagglutinin activities of *P. gingivalis* were evaluated with continuously diluted bacterial solution (from left to right) and incubated with sheep erythrocytes (2%) in a 96-well plate with a U-shaped bottom. The red arrow indicates the maximum dilution multiple of complete agglutination, that is, the erythrocyte agglutination titer. Changes in the HagA expression of *P. gingivalis* in single- and dual-species biofilm under a **(B)** low- and **(C)** high-heme environment. ^***^p < 0.001; NS *p* > 0.05. LH, low heme; HH, high heme; Coc, coculture; and Mon, monoculture.

We explored the ability of *P. gingivalis* to agglutinate erythrocytes, which can reflect the virulence and the degree of absorption of heme of *P. gingivalis*. As shown in Figure 5A, the first row was *P. gingivalis* in the dual-species biofilm, with a titer of 16, while the titer of the second row was 4, which represented *P. gingivalis* in a single-species biofilm. *Porphyromonas gingivalis* in the dual-species biofilm showed a stronger ability to agglutinate erythrocyte. When there was an excess of heme in the culture, the titers of *P. gingivalis* in the two types of biofilms were both two.

The PCR results showed that after the formation of biofilms with *C. albicans*, the expression of HagA in *P. gingivalis* was more than three times higher than that in the heme-restricted environment alone, and this difference disappeared under the condition of sufficient heme (Figures 5B,C).

### The Expression of Genes Related to Heme Utilization in *Porphyromonas gingivalis*

**Figure 6:**
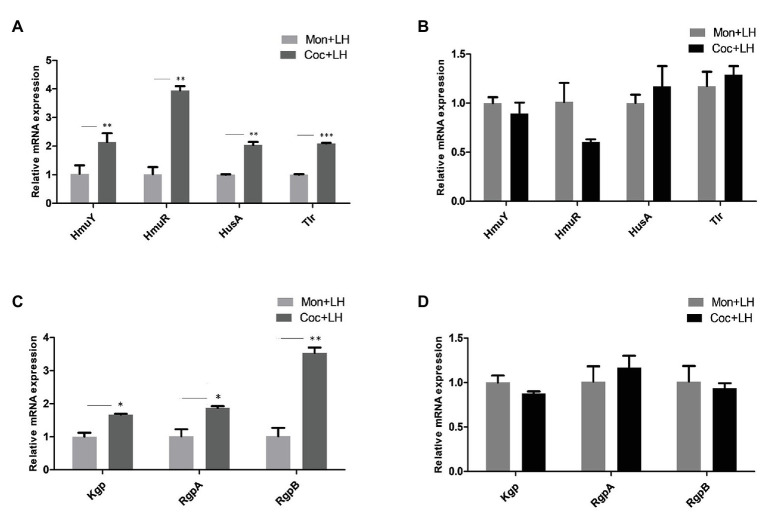
The expression of mRNA related to heme utilization in *P. gingivalis*. Gene expression for heme utilization in *P. gingivalis* cocultured and monocultured for 24 h with **(A,C)** low heme and **(B,D)** high heme, respectively. ^*^p < 0.05; ^**^p < 0.01; ^***^p < 0.001. LH, low heme; HH, high heme; Coc, coculture; and Mon, monoculture.

To further uncover the molecular mechanism of changes of *P. gingivalis* in single- and dual-species biofilms, we assessed the gene expression levels related to heme utilization. In the dual-species biofilm, the expression of HmuR, a TonB-dependent receptor, increased nearly three times, and the expression of HmuY, HusA, and Tlr, were also more than twice as high as that in single-species biofilms (Figure 6A).

The genes encoding gingipains (Kgp, RgpA, and RgpB), which are involved in red blood cell adhesion and hemoglobin hydrolysis, were also highly expressed to varying degrees in the dual-species biofilm (Figure 6C). When the heme concentration in the culture solution was increased to 5 μg/ml and the heme competition between *C. albicans* and *P. gingivalis* disappeared, there were no significant differences between the genes of *P. gingivalis* in the two types of biofilms (Figures 6B,D). In summary, *P. gingivalis* occupied a superior position in the nutrient competition with *C. albicans* by increasing the gene levels related to hemoglobin utilization.

## Discussion

The oral cavity is the site with the most microbial species in the human body, in which a large number of bacteria and fungi exist (Arweiler and Netuschil, 2016; Baker et al., 2017). Various microorganisms are distributed in different niches in the oral cavity, such as tooth surfaces, the mucosal surface, or the gingival sulcus. With an aging population and the enhancement of oral health care awareness, the population of partial denture wearers has increased, while about two thirds of denture wearers suffer from denture stomatitis (Tobouti et al., 2016). At the same time, data showed that there is more frequent occurrence of subgingival *C. albicans* in periodontitis patients wearing dentures as compared to healthy people, which inevitably affects other subgingival microorganisms (Marsh and Zaura, 2017).

Nutritional competition exists in various microbial communities. Different from nutrient-rich culture medium, the oral cavity contains only scarce nutrient elements, and there is fierce competition between microorganisms for nutrition, such as the competition between *Streptococcus gordon* and *Streptococcus mutans* for amino sugar in saliva and the competition between *Streptococcus sanguis* and *S. mutans* for D-alanine (Kreth et al., 2005; Zeng et al., 2016). *Porphyromonas gingivalis* and *C. albicans* can be detected in subgingival sites at the same time, and both of them need to utilize heme to meet their growth needs. However, there are few studies on whether there is heme competition between *P. gingivalis* and *C. albicans* and what comprises their specific competitive pathways. In order to clarify these problems, we first compared the changes in *P. gingivalis* in the single- and dual-species biofilms under heme restriction conditions, and then provided a high-heme environment to carry out reverse verification. The results confirmed that *P. gingivalis* competed with *C. albicans* for heme, and the existence of *C. albicans* posed a threat to the stability of the subgingival microenvironment.

When different microorganisms are in the same niche, there can be synergy, protection, killing, or even coexistence of multiple relationships (Hansen et al., 2007; Harrison et al., 2008; Garbeva et al., 2011). Previous studies found that the competition for iron sources led to the death of *C. albicans*, while protecting *P. aeruginosa* to some extent (Trejo-Hernandez et al., 2014). Previous studies have shown that *C. albicans* can protect anaerobic bacteria by reducing the oxygen concentration in biofilms (Fox et al., 2014; Bartnicka et al., 2019), and therefore, an hypoxic environment was selected as the culture condition because, it is similar to what is observed in clinical practice. In the current study, the existence of *C. albicans* increased the viable *P. gingivalis* in the dual-species biofilm, but after 36 h, there was a decrease in living *C. albicans*. In the high-heme environment, this difference disappeared, and the number of living *C. albicans* significantly increased (Figures 1, 2).

The survival of the fittest is a very common phenomenon as microorganisms compete for nutrition (Marsh and Zaura, 2017). The winning bacterial population can often more effectively absorb resources from the environment and use a series of killing mechanisms, such as secretion of bacteriocin or change in the oxygen content in the environment, thus damaging competitors (Kreth et al., 2005; Trejo-Hernandez et al., 2014). Our results indicated that *P. gingivalis* and *C. albicans* can compete for heme in the environment when heme is restricted. *Porphyromonas gingivalis* occupies a superior position and can use various methodologies to kill *C. albicans*. However, when heme is sufficient, the number of dead *C. albicans* decreased, which may suggest that the competition for heme led to a decrease in *C. albicans* activity (Figures 2A,C). Therefore, the availability of heme directly affects the population dynamics of *P. gingivalis* and *C. albicans*.

Compared with the floating state, the microorganisms in biofilm can more effectively resist external pressure due to the encapsulation of EPS, and have unique advantages in metabolism, acid-base regulation, and other aspects (Peleg et al., 2010; Kim et al., 2018; Wong et al., 2018; Caro-Astorga et al., 2020). We found that when heme is limited, a large amount of EPS was observed in the dual-species biofilm, while in the single-species biofilm of *P. gingivalis*, there were fewer EPS and bacteria. When heme is abundant, these differences disappeared, and we observed approximately equal amounts of EPS and microorganisms in the both biofilms (Figure 3). All results suggested that when heme competition exists in the biofilm, more EPS was produced. EPS provides satisfactory protection for microorganisms in biofilm, which enables microorganisms to more effectively resist external stimuli (Sharma et al., 2016; Pang et al., 2017). *Porphyromonas gingivalis* with normal morphology in dual-species biofilm also verified this conjecture, while *P. gingivalis* in single-species biofilm without EPS protection exhibited an abnormal elongated rod shape.

The complement system in the host plays a crucial role in preventing bacterial infection, and resisting serum killing is also one of the virulence characteristics of *P. gingivalis* (Slaney et al., 2006; Popadiak et al., 2007). We found that under the condition of low heme, *P. gingivalis* co-cultured with *C. albicans*, can more effectively escape being killed by serum (Figure 4C). Gingipain is an important secreted protein of *P. gingivalis*. Studies have shown that gingipain can damage host defense molecules and degrade complement in blood, thus providing assistance to *P. gingivalis* to escape the immune-killing effect (Glowczyk et al., 2017). The PCR results in Figure 6C show that the expression of the Kgp, RgpA, and RgpB genes encoding gingipains were significantly increased in the dual-species biofilm in the low-heme environment, which can explain the higher survival rate of *P. gingivalis* in the serum escape experiment. In addition, *P. gingivalis* in the dual-species biofilm was more resistant to the killing effect of cefazolin and sulfamethoxazole. These differences in virulence cease to exist when heme is abundant. The sum of these observations suggests that the competition for heme promotes the expression of virulence of *P. gingivalis*.

Further exploration is necessary to determine which pathway *P. gingivalis* utilizes to increase its advantage in heme competition. Different from other Gram-negative bacteria, *P. gingivalis* cannot produce ironophore and needs to use specific outer membrane receptors, proteases, and lipoproteins to obtain iron or heme from the outside (Olczak et al., 2005). Dashper et al. (2009) found that when the concentration of heme is low, the expression of proteins related to heme utilization of *P. gingivalis* increases. Therefore, we suspect that competition with *C. albicans* for heme will promote further high expression of genes related to heme utilization by *P. gingivalis*. In addition to detecting the classical hmu family genes (HmuY, HmuR), we also analyzed HusA and Tlr, which respectively represent the other two pathways for *P. gingivalis* to absorb heme (Yukitake et al., 2011; Smalley and Olczak, 2017). We found that the expression of HmuY and HmuR in the dual-species biofilm increased by two times and four times, respectively (Figure 6A). HmuY is a lipoprotein that can bind with heme and is responsible for the transfer of heme (Carvalho-Filho et al., 2016; Slezak et al., 2020). HmuR is a Tonb-dependent receptor that can absorb the heme transferred by HmuY into the bacteria (Stebbins et al., 2009). The upregulation of these two genes indicates that *P. gingivalis* activates the hmu family and increases heme utilization efficiency in the process of competing with *C. albicans* for heme. Additionally, the expression of HusA and Tlr also significantly increased, suggesting that these two heme utilization pathways may also assist *P. gingivalis* in obtaining heme. As for the expression of other genes on these two pathways, further verification is needed.

The hmu family also needs to cooperate with gingipains to utilize heme. Gingipains include lysine-specific proteases (Kgp) and arginine-specific proteases [Rgps(RgpA and RgpB); Smalley and Olczak, 2017]. The adhesion regions of Kgp and RgpA participate in the adhesion of red blood cells, while the decomposition of red blood cells and the hydrolysis of hemoglobin mainly depend on the hydrolysis regions of Kgp, RgpA, and RgpB (Smalley et al., 2007; Li et al., 2011). In addition, the gingipains of *P. gingivalis* can interact with the surface proteins of *C. albicans* to promote the stability of the dual-species biofilm, and can also regulate the host reaction by hydrolyzing the main defense molecules (Bartnicka et al., 2019, 2020). Therefore, gingipains play an important role in the perpetuation of biofilms. In the current study, the mRNA expression of Kgp, RgpA, and RgpB in the dual-species biofilm was higher than that in the single-species biofilm, suggesting that the adhesion and hydrolysis activity of *P. gingivalis* may be significantly increased after co-culture with *C. albicans* when heme was limited (Figures 6C,D). This is also consistent with the agglutination of red blood cells observed in Figure 5A.

Participating in the utilization of heme, the gingipains are also one of the major secreted proteins of *P. gingivalis* and are an important virulence factor (de Diego et al., 2014; Santos-Lima et al., 2019). Imamura et al. (1994) reported that HRgpA and RgpB can accelerate the formation of gingival crevicular fluid (GCF) and the progression of inflammation. O’Brien-Simpson also confirmed that the RgpA-Kgp complex in gingipains is related to the death of epithelial cells, fibroblast, and endothelial cells, leading to inflammation, tissue destruction, and vascular rupture (hemorrhage; O’Brien-Simpson et al., 2009). Under the condition of heme restriction, the expression of RGPs and Kgp increased, which may not only enhance the efficiency of heme intake, but also raise the concentration of heme in the environment in some destructive ways. In addition, gingipains can also cause the apoptosis of osteoblasts and accelerate the absorption of alveolar bone (Qiu et al., 2018). Therefore, due to the presence of *C. albicans*, *P. gingivalis* was able to increase its ability to destroy periodontal tissues and accelerate the progress of periodontal disease.

When the heme concentration increased to 5 μg/ml, the heme competition between *P. gingivalis* and *C. albicans* ceased, and there were no significant differences in mRNA levels of heme utilization genes and gingipains between single- and dual-species biofilms (Figures 6B,D). We can speculate that increased genes of *P. gingivalis* caused by heme competition are likely to occur in the early stage of periodontitis, when the concentration of heme in the gingival sulcus is relatively low. When the concentration of heme increases, a nutrient-rich environment is formed in the gingival sulcus, which promotes the growth of microorganisms (Fyrestam and Ostman, 2017; Roy and Kornitzer, 2019). This also explains the conflict between the previously observed decrease in viable *C. albicans* (Figures 1, 2) and the increased frequency of subgingival *C. albicans* isolated from patients with actual periodontitis (Urzua et al., 2008; McManus et al., 2012).

In summary, this study indicates that the pathogenic potential of *P. gingivalis* was enhanced by *C. albicans* through heme competition. The increased expression of genes related to heme utilization is the reason for its dominant position in the competitive relationship, and it is more likely to promote the occurrence and development of periodontitis. Additional research on *C. albicans* will be necessary to determine the effect of heme, an important nutrient, on the interaction between *P. gingivalis* and *C. albicans*.

## Data Availability Statement

The raw data supporting the conclusions of this article will be made available by the authors, without undue reservation.

## Author Contributions

YG: data curation, formal analysis, investigation, methodology, writing-review, and editing. YuW: formal analysis, investigation, and methodology. YiW: investigation and supervision. YJ: writing-review and editing. CW: funding acquisition, project administration, resources, writing-review, and editing. All authors contributed to the article and approved the submitted version.

### Conflict of Interest

The authors declare that the research was conducted in the absence of any commercial or financial relationships that could be construed as a potential conflict of interest.
