# Supplementary material for: Heme Competition Triggers an Increase in the Pathogenic Potential of Porphyromonas gingivalis in Porphyromonas gingivalis-Candida albicans Mixed Biofilm
Source: Front Microbiol. 2020 Dec 2;11:596459. doi: 10.3389/fmicb.2020.596459 (PMC7738433; doi:10.3389/fmicb.2020.596459)
Supplement: Supplementary file 1 [file Data_Sheet_1.pdf]

**Supplemental Materials**

1. *Separation of P. gingivalis and C. albicans*

The microbes were separated by Percoll gradient centrifugation (Percoll, Sigma Aldrich, P1644). The four stock solutions of 50, 60, 70 and 80% Percoll were prepared with PBS, and 2ml of each solution was manually layered after wetting the centrifuge tube with FBS. The mixed biofilm of *P. gingivalis* and *C. albicans* was collected, dissolved in 2 ml of ice-cold stop buffer, and ultrasonically oscillated for 10 s. The solution of dual-species biofilm was placed at the top of the gradients, which were centrifuged at 300 ×*g* for 5 min and 8,000 ×*g* for 10 min using a fixed-angle rotor at 4 °C. Finally, the top (*P. gingivalis*) and lower (*C. albicans*) fractions were collected and washed twice with 5ml PBS.

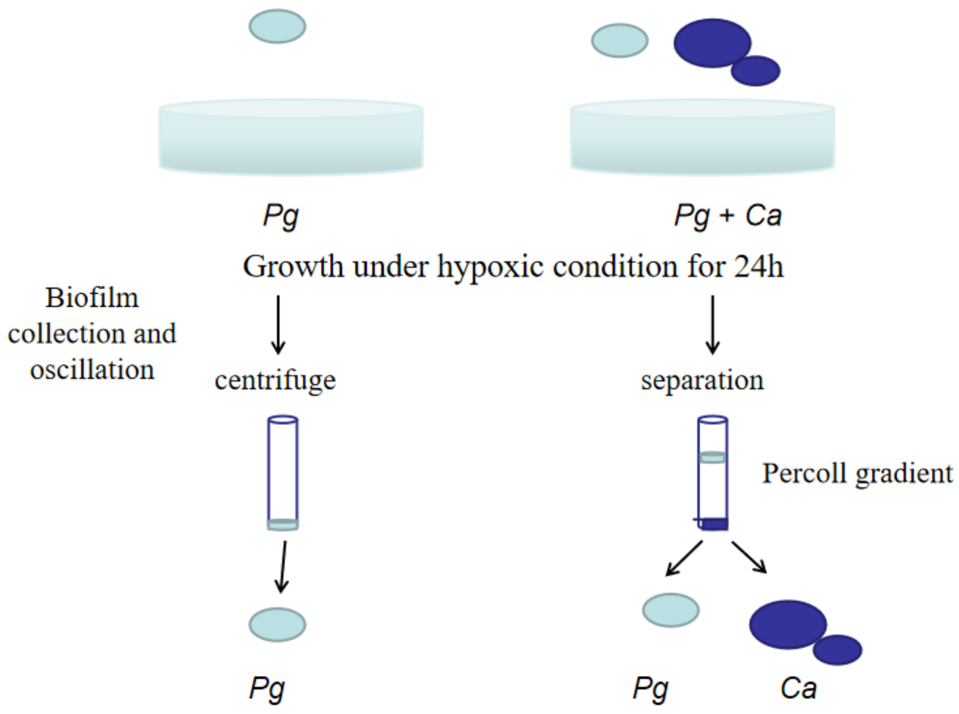


2. *Gram stain of C. albicans and P. gingivalis*

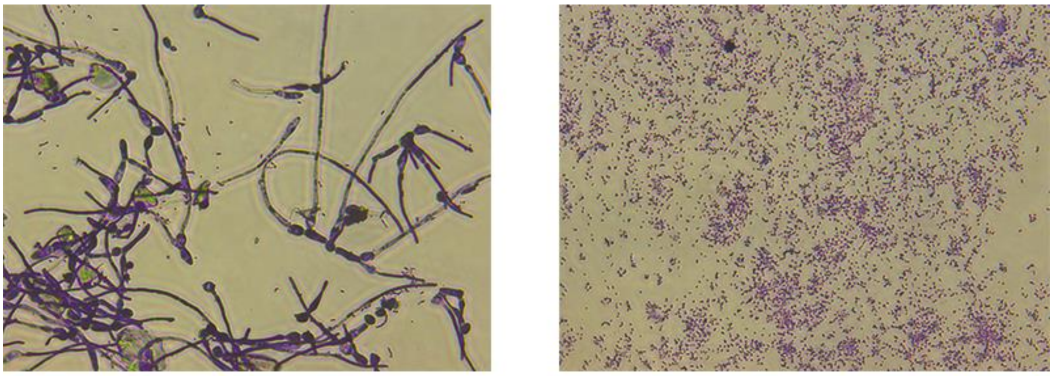

*C. albicans* *P. gingivalis*

Microoranisms were separated from the dual-species biofilm and stained with Gram. Microscopic images of *C.albicans* (Left) and *P. gingivalis* (Right) were shown above.

*3. Statistical data of Candida albicans*

In Figure 1A, CFU of *C. albicans* decreased at 36h and 48h, with statistical difference （$P < 0.05$）.

| Time | Mean ± s.d | P |
|---|---|---|
| 36h | 6.812 ± 0.027 | P=0.0299 |
| 48h | 6.652 ± 0.034 | |

*4. CFU of P. gingivalis after serum treatment*

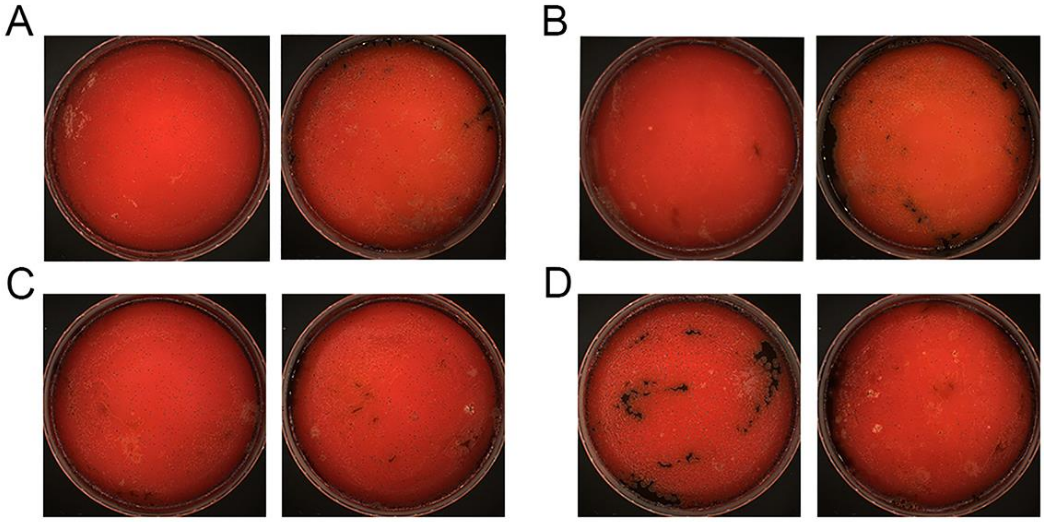


*P. gingivalis* separated from biofilms were treated in serum and cultured on BHI plates for 7 days. Growth of *P. gingivalis* treated with heat-inactivated serum（left） or serum from healthy volunteers （right）. (A) *P. gingivalis* in dual-species biofilm under low heme condition. (B) *P. gingivalis* in single-species biofilm under low heme condition. (C) *P. gingivalis* in dual-species biofilm under high heme condition. (D) *P. gingivalis* in single-species biofilm under high heme condition.
